# The "Medical Press" and the Hospital Sunday Fund

**Published:** 1897-09-11

**Authors:** 


### THE "MEDICAL PRESS" AND THE HOSPITAL SUNDAY FUND.

The *Medical Press and Circular*, in defending the position it has chosen to take up in regard to the Hospital Sunday Fund, accuses us of misquotation, and does so in such a way and with such a fine show of indignation as to suggest that our trivial error, apparently an error in dictation, was of some importance to the argument. "We defy the *Hospital*," says the *Medical Press and Circular*, "to show that we printed those words." The words in question are: "Does the Hospital Sunday Fund make any attempt whatever to regulate its grants in proportion to the wise and economical expenditure of individual charities? On the contrary, its chief condition is the relation of administration to maintenance, a fallacious condition, that simply supplies a direct incentive to reckless expenditure." We refer the *Medical Press* to a leading article on page 114 of its issue of August 4th last, line 43, *et seq.* It is there printed: "Does the Hospital Sunday Fund make any attempt whatever to regulate its grants according to the wise and economical expenditure of individual charities? On the contrary, . . . its chief *test* is the relation of administration to maintenance, a fallacious condition that simply supplies a direct incentive to reckless expenditure." We are sorry for the errors, which are mere trifles and obviously accidental, but we think that the *Medical Press and Circular*, instead of speaking of them as misquotations and so leading its readers to think they were of some importance, should have either given the exact words or have explained that the alteration touched neither the argument nor the sense.

